# Prepared Gold for Filling Teeth

**Published:** 1853-10

**Authors:** 


### Editorial.

### PREPARED GOLD FOR FILLING TEETH.

We have recently seen specimens of this preparation for filling teeth, prepared by Dr. H. R. White & Co., Utica, N. Y. We have seen some specimens of fillings out of the mouth with this article, which look very well; and in one which we had an opportunity of examining, there appeared to be a most perfect adaptation of the gold to all the inequalities of the cavity. It is introduced much in the same manner as amalgam, being sufficiently soft and tenacious, or adhesive, to be introduced in the same way, and by pressure consolidated. We take this opportunity to acknowledge the receipt of a specimen of prepared gold by Drs. Taft & Watt of Xenia—we believe of their own manufacture—which, so far as we can judge, will answer a good purpose. Dr. Taft writes us that he uses it almost exclusively for plugging, and considers it an important improvement in the preparation of gold for filling teeth.

Dr. J. M. Brown is the agent for White & Co.'s prepared gold, and can supply the profession with the article.

